# Is the High Proportion of Males in a Population of the Self-Incompatible *Fraxinus platypoda* (Oleaceae) Indicative of True Androdioecy or Cryptic-Dioecy?

**DOI:** 10.3390/plants11060753

**Published:** 2022-03-11

**Authors:** Hitoshi Sakio, Takashi Nirei

**Affiliations:** 1 Sado Island Center for Ecological Sustainability, Niigata University, Sado 952-2206, Japan; 2 Saitama Museum of Natural History, Nagatoro 369-1305, Japan; ulmus201@gmail.com

**Keywords:** androdioecy, andromonoecious tree, diallelic self-incompatibility (DSI), dioecy, *Fraxinus platypoda*, male tree, pollen germination, seed development, sexual expression

## Abstract

Androdioecy is a rare reproductive system. *Fraxinus platypoda*, a woody canopy species in Japan’s mountainous riparian zones, is described as a morphologically androdioecious species. In this study, we tried to detect whether *F. platypoda* is also functionally androdioecious. We analyzed its sexual expression, seed development, pollen morphology and germination ability, pollination systems, and mast flowering behavior. We found that the hermaphrodite trees are andromonoecious, with inflorescences bearing male and hermaphroditic flowers, whereas male individuals had only male flowers. Pollen morphology was identical in male flowers, in hermaphrodite flowers of an andromonoecious individual, and in male flowers of male individuals. Pollen from both types of individuals was capable of germination both ex vivo (on nutrient medium) and in vivo in pollination experiments. However, compared with pollen from andromonoecious trees, pollen from male trees showed a higher germination rate. The self-pollination rate of bagged hermaphroditic flowers was almost zero. The fruit set rate following cross-pollination with male pollen from a male tree was higher than that following natural pollination, whereas the rate with hermaphroditic pollen was the same. The flowering and fruiting of *F. platypoda* have fluctuated over 17 years; the flowering of the two types of sexual individuals exhibited clear synchronization during this period. The frequency of male individuals within the populations is 50%. The maintenance of such a proportion of males in populations of the self-incompatible *F. platypoda* is either indicative of a true androdioecious species with a diallelic self-incompatibility system or a cryptic-dioecious species. This alternative is discussed here.

## 1. Introduction

Comparisons of the diverse sexual expressions of plants are important for understanding plant evolution. Various types of sexual expression are found in flowering plants [1]. In hermaphroditism, the most common type, flowers possess both female and male reproductive organs. In contrast, dioecious plants comprise separate female and male individuals. Gynodioecy and androdioecy are intermediate evolutionary states between bisexual and monosexual conditions [2,3], with androdioecy being one of the rarest sexual expressions [4,5,6,7,8]. Morphological androdioecy includes both functional dioecy and functional androdioecy. In functionally dioecious plants exhibiting morphological androdioecy, the anthers of hermaphroditic flowers are indehiscent or the pollen grains are inviable, and the flowers are female. In functional androdioecy, in contrast, viable pollen is produced in hermaphroditic flowers [9].

To date, functional androdioecy has been reported in only a few plants. Among herbaceous plants, *Oxalis suksdorfii* Trel. [10], *Datisca glomerata* Baill. [11], *Mercurialis annua* L. [12], *Schizopepon bryoniifolius* Maxim. [13], and *Fritillaria persica* L. [14] are functionally androdioecious. Shrubs exhibiting functional androdioecy include *Phillyrea angustifolia* L. [3,15,16,17,18,19], *Osmanthus delavayi* Franch. [20], *Osmanthus fragrans* Lour. [21], and *Morinda umbellata* L. ssp. *boninensis* T.Yamaz. [22]. Among sub-tall trees (10 m < height < 20 m), *Fraxinus lanuginosa* Koidz. [23], *Fraxinus sieboldiana* Bl. [24], and *Gymnocladus assamicus* Kanjilal ex. P.C. Kanjilal [25] are functionally androdioecious, and the same is true for the following tall trees (20 m < height < 30 m): *Tapiscia sinensis* Oliv. [26], *Fraxinus ornus* L. [19,27], *Fraxinus longicuspis* Sieb. et Zucc. [28], and *Chionanthus retusus* Lindl. & Paxton [29]. Due to obvious observational and experimental difficulties, no reports of functional androdioecy have appeared in trees taller than 30 m.

*Fraxinus platypoda* Oliv. (Oleaceae) is a deciduous woody canopy species that can reach up to 40 m in height and over 150 cm in diameter at breast height [30]. The maximum lifetime of *Fraxinus platypoda* individuals is over 250 years [31]. This species is a late successional species in riparian zones of cool temperate forests distributed along the Pacific coast of Japan [32]. There are 48 species of *Fraxinus* distributed in the northern hemisphere, and *F. platypoda* is unclassified due to its uncertain position in the phylogenetic tree [33]. *F. platypoda* is morphologically androdioecious, with male and hermaphroditic individuals in the population [30]. As described above, it has not been confirmed whether *F. platypoda* is morphologically and functionally androdioecious.

In this study, we investigated the morphological and functional sex expression of *F. platypoda* and examined its reproductive strategies. We revisited the sexual morphology between individuals, and thus, we examined the functionality of an androdioecious breeding system. Then, we observed the seed developmental process from flowers to seeds and compared the morphology and germination ability of pollen grains between males and hermaphrodites. Pollination experiments were also performed. On the basis of our results, we discuss the question of the maintenance of such a system: the frequency of male individuals (50%) suggests that it is either a crypto-dioecious species or, more probably, a diallelic self-incompatible (DSI) species.

## 2. Materials and Methods

### 2.1. Materials

*Fraxinus platypoda* is distributed from Tochigi Prefecture in the north to Miyazaki Prefecture in the south [32], and dominates forests in riparian zones [31]. The sexual expression of *F. platypoda* is morphologically androdioecious [30]. The flowers bloom from mid-April to early May, but the time of blooming varies with elevation. Flower buds form on both sides of the apical bud of branches that have passed through the winter. Male and hermaphroditic flowers bloom almost simultaneously, but male flowers tend to bloom a few days earlier than hermaphroditic ones. The species is wind-pollinated (anemophilous) and anemochorous.

### 2.2. Study Sites

**Figure 1:**
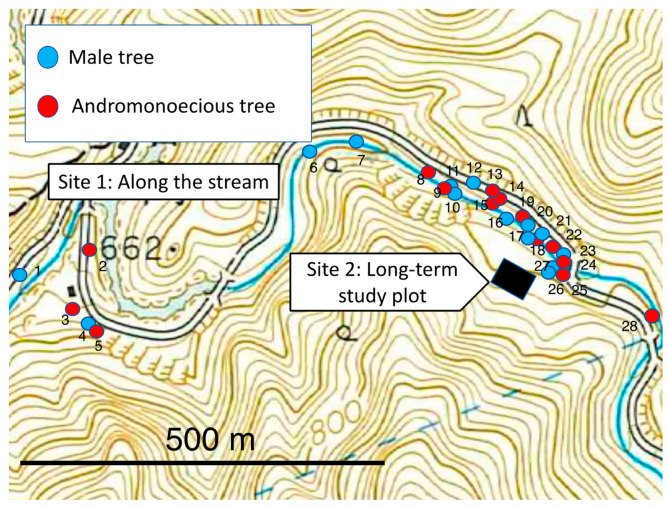
Site 1: distribution of *Fraxinus platypoda* mature trees along the stream. Location: Chichibu Mountains, Saitama Prefecture, Japan (35°55′ N, 138°59′ E, 650 elevation). Site 2: black rectangle shows the location of the long-term study plot.

Site 1: The population was distributed over approximately 1 km along a stream and consisted of 28 mature trees. The site was located in the Ohchigawa section of the University of Tokyo Chichibu Forest (UTCF: Figure 1) in the Chichibu Mountains, Saitama Prefecture, Japan (35°55′ N, 138°59′ E, 650 m elevation). The mean annual temperature at the study site is 9.7 °C, with a mean air temperature of 21.0 °C in the warmest month (August) and −1.6 °C in the coldest month (January). The mean annual precipitation at the study site was 1616 mm from 1990 to 2006 [34].

**Figure 2:**
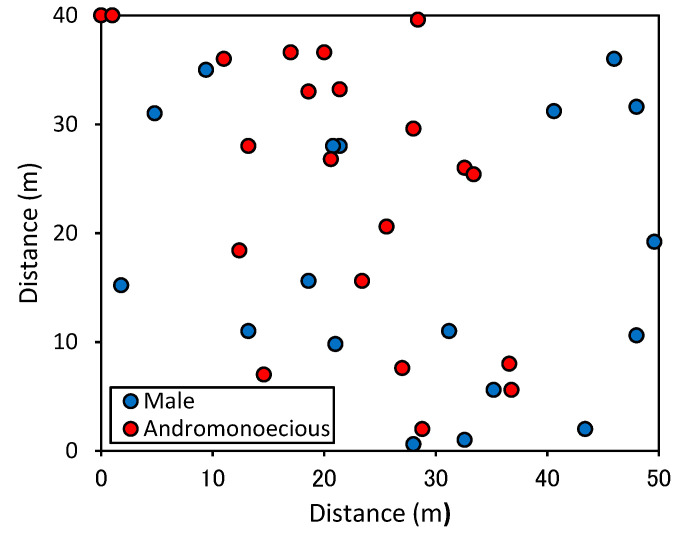
Site 2: distribution map of mature *Fraxinus platypoda* trees within the rectangular long-term study plot (50 × 40 m). Blue and red circles indicate 18 male and 21 andromonoecious mature trees, respectively.

Site 2: The population was located in a rectangular plot (50 × 40 m, the long-term study plot) in the UTCF (see Figure 2) and consisted of 39 mature trees.

Site 3: The natural forest at Urayama (35°52′ N, 139°06′ E, 790 m elevation) located 12 km away from the UTCF. In this forest, three trees were selected: one recipient experimental tree (X) and two donor trees; a male tree (Y) and an andromonoecious tree (Z).

### 2.3. Methods

#### 2.3.1. Floral Morphology

##### Sexual Phenotypes

At site 1, the floral morphology of inflorescences of adult male and hermaphroditic trees was observed as follows. First, six, five, and five branches were cut from each of three adult hermaphroditic trees in the Ohchigawa section of the UTCF on 16 April 2009. We checked the sexual status (male or hermaphroditic) of flowers in 60 inflorescences from the morphologically hermaphroditic trees. In addition, we measured the pistil lengths of 20 hermaphroditic flowers per tree. On 15 April 2013, we cut 10 branches from one male adult tree and 12 branches from morphologically hermaphroditic trees at the UTCF study site and checked the sexual status of their inflorescences. On 16 April 2021, we cut six branches from one male adult tree and eight branches from a morphologically hermaphroditic tree at the UTCF study site and checked the sexual status.

At site 2, we also observed the long-term dynamics of flowering and sexual phenotypes. All the trees in this plot had bloomed at least once in 17 years. To observe the long-term dynamics of flowering and fruiting, a 40 m × 50 m study plot was established to monitor the experimental individuals in 1990 (Figure 2). We measured the DBH (diameter at breast height) of all the trees and mapped their distribution in the plot.

##### Seed Development

The seed development of hermaphroditic flowers of adult trees was observed at UTCF in 1992. *F. platypoda* has winged fruits (samaras) with seeds inside the pericarp. Individual inflorescences (one per tree) were collected 13 times from 8 May to 27 October. After length and width measurements, pericarps and seeds were dried at 80 °C for 48 h and weighed.

#### 2.3.2. Pollen

##### Pollen Morphology

Pollen was collected on 19 April 1996, from male and hermaphroditic flowers at the study site. To identify morphological differences between male and hermaphroditic flowers, 30 pollen grains of each type were subjected to acetolysis, mounted in glycerin jelly, and measured under an optical microscope. Differences in polar length, equatorial diameter, and exine thickness were assessed using the Kruskal–Wallis test followed by Steel–Dwass multiple comparison.

##### Pollen Germination Ability

Pollen from hermaphroditic and male adult trees was collected at the UTCF study site on 21 April 1995. Mature pollen grains were cultured at 20 °C for 48 h in the dark on solid medium containing 10% sucrose and 1% agar from 24–26 April 1995. To determine the pollen germination rate, 100 pollen grains were counted under a Zoom binocular stereomicroscope. A pollen grain was considered to have germinated when its pollen-tube length was longer than the diameter of the pollen grain. The experiment was repeated five times with each of the two types of flowers.

#### 2.3.3. Pollination Experiments

We carried out four types of pollinations: (i) cross-pollination with pollen from male trees; (ii) cross-pollination with pollen from hermaphrodite trees; (iii) self-pollination; and (iv) open pollination with pollen from the whole population. At the beginning of April when the flower buds began to swell, branches of the recipient trees were selected randomly and covered with bags for pollination types (i), (ii), and (iii). The branches were not covered with bags for pollination type (iv)—this allowed for open pollination, and eventually, fruit set. At this time, the surrounding trees were not in bloom and no pollen was being dispersed. In parallel, we collected pollen from donor trees in the laboratory. Flowers on branches of the donor trees were opened in the laboratory; then, pollen was collected from the anthers. The branches of the different pollen donor trees were kept separate to avoid mixing of pollen.

On a windless day in late April, the bags were removed from recipient trees for pollination types (i) and (ii). For types (i) and (ii), the bags were opened before hand pollination using pollen from a male tree or pollen from a hermaphrodite tree that had already been collected and stored in the laboratory. Notably, during hand pollination, the pollen present in the air (it is an anemophilous species) was a potential contaminant that could modify the results of the crossing tests. For pollination type (iii), the bags remained unopened from the time they were placed (before the blossoming of the population) until mid-May. All bags were removed in mid-May, when pollen dispersal had finished and pollination of flowers on surrounding trees was complete. The results of these crossing experiments conducted over multiple years are shown in Table 2.

## 3. Results

### 3.1. Morphological Characteristics

#### 3.1.1. Sexual Phenotypes

##### Frequencies of Observed Sexual Phenotypes: Description of Both Populations

In both populations, the hermaphroditic individuals were in fact all andromonoecious, with both hermaphroditic flowers and male flowers in their inflorescences. However, there was no gradual variation in sexual phenotypes. This differed from the polygamous situation in *Fraxinus excelsior* [35], where the sexual phenotypes range from totally male to hermaphroditic (inflorescences with a range of proportions of male and female flowers) to totally female.

At site 1, the population was distributed over approximately 1 km along the stream consisted of 14 male mature trees and 14 andromonoecious mature trees (=28 trees, see Figure 1). At site 2, the population in the long-term 50 × 40 m rectangular study plot consisted of 18 male trees and 21 andromonoecious trees (Figure 2). At both sites, the frequency of each of the two sexual phenotypes (male trees and andromonoecieous trees) was about 50%.

##### Flower Morphology on the Basis of Flower Type

**Figure 3:**
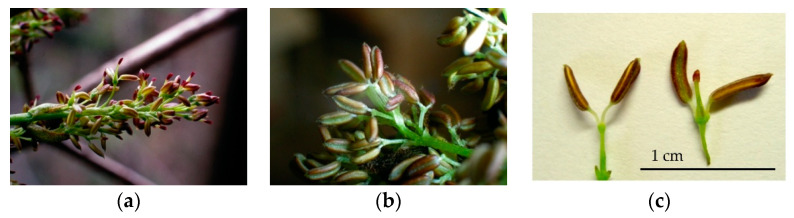
Flower types of *Fraxinus platypoda*. (**a**,**b**) Inflorescences of andromonoecious (**a**) and male (**b**) trees. (**c**) Male (left) and hermaphroditic (right) flowers.

On the basis of flower type, *F. platypoda* trees fall into two categories: male trees with only male flowers (Figure 3b,c), and andromonoecious trees with both male flowers and hermaphroditic flowers within a single inflorescence (Figure 3a,c). Both types of flowers lack a corolla. A single inflorescence (panicle) is composed of dozens or hundreds of flowers of either type. Male flowers have a single stamen with a pair of anthers, whereas hermaphroditic flowers have one pistil with a pair of anthers (Figure 3c). In both types of trees, flowers in inflorescences open at approximately the same time; according to our observations, however, male trees tended to bloom a few days earlier than andromonoecious trees. We found that the anthers of both types of flowers opened spontaneously and released pollen, which indicated that both flower types have dehiscent anthers.

**Figure 4:**
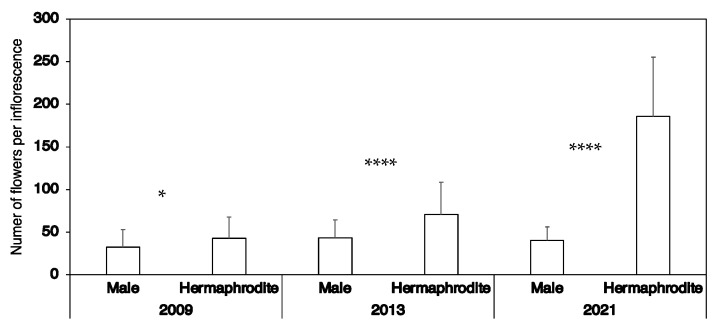
The number of hermaphroditic and male flowers per inflorescence on andromonoecious trees in 2009, 2013 and 2021. Bars indicate standard error. *, *p* < 0.05; ****, *p* < 0.0001.

In 2009, all 60 examined inflorescences on andromonoecious trees had both hermaphroditic and male flowers. The number of flowers per inflorescence was 75.4 ± 29.3, of which 32.6 ± 20.4 were male and 42.8 ± 24.9 were hermaphroditic (Figure 4). The number of hermaphroditic flowers per inflorescence on andromonoecious trees was significantly higher than the number of male flowers (Student’s *t*-test, *p* < 0.05). The average pistil length was 3.7 ± 0.8 mm and ranged from 1.4 mm to 4.8 mm. In 2013, all 40 inflorescences on andromonoecious trees also had hermaphroditic and male flowers. The number of flowers per inflorescence was 113.9 ± 52.6, with 43.3 ± 21.3 male and 70.6 ± 38.3 hermaphroditic flowers. In 2021, the number of hermaphroditic and male flowers per inflorescence showed a similar trend to that in 2009 and 2013 (Figure 4). The number of hermaphroditic flowers per inflorescence was significantly higher than that of male flowers (Welch’s *t*-test, *p* < 0.0001). Forty (2013) and twenty (2021) inflorescences on male trees had only male flowers. The number of flowers per inflorescence on observed male trees in 2013 and 2021 was 112.9 ± 41.4 and 142.4 ± 43.0, respectively.

These results show that there are two sexual phenotypes of *F. platypoda* trees: male trees with male flowers only, and andromonoecious trees with hermaphroditic flowers and male flowers in the same inflorescence.

#### 3.1.2. Seed Development

**Figure 5:**
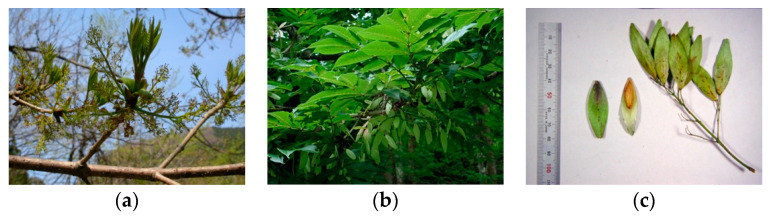
Fruit development in *Fraxinus platypoda*. (**a**) Small fruits after flowering on 28 April. (**b**) Fruits with no seeds on 12 June. (**c**) Seeds in pericarps in autumn.

**Figure 6:**
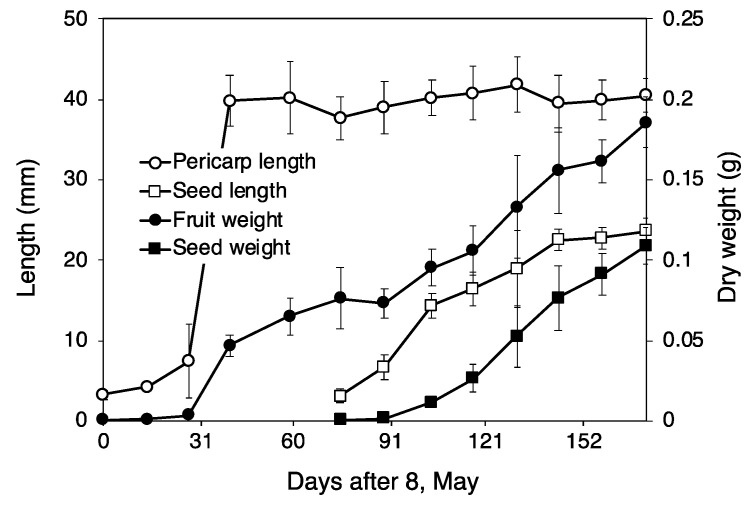
Fruit and seed development in *Fraxinus platypoda*. Bars indicate standard error.

*F. platypoda* has winged fruits. The seeds, which are approximately 2 cm in length, are enclosed in 4 cm pericarps (Figure 5c). In this study, fruits of *F. platypoda* formed immediately after flowering and continued growing until mid-June (Figure 5a,b). Seeds began increasing in size in mid-July and ceased expansion in late September. Seed dry weight continued to increase until late October. Pericarp formation in *F. platypoda* ceases by early summer; this process is followed by seed formation, which is completed in autumn (Figure 6).

### 3.2. Pollen

#### 3.2.1. Pollen Morphology

**Figure 7:**
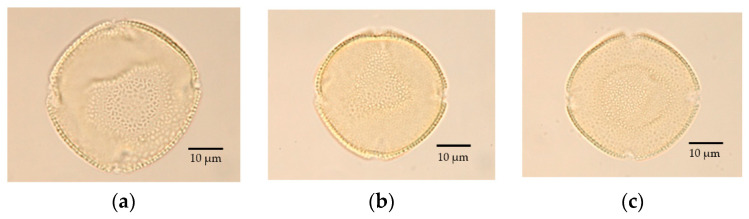
Pollen morphology of *Fraxinus platypoda* in polar view. (**a**–**c**) Pollen grains from a male flower of a male tree (**a**), a male flower of an andromonoecious tree (**b**), and a hermaphroditic flower of an andromonoecious tree (**c**).

On the basis of the sexual expression of *F. platypoda*, pollen was divided into three types: pollen from male flowers of male trees (M), pollen from male flowers of andromonoecious trees (MA), and pollen from hermaphroditic flowers of andromonoecious trees (HA) (Figure 7). In polar view, pollen grains were mostly semi-angular to circular and were mainly oblate-spheroidal when viewed equatorially (polar length/equatorial diameter = 0.91, 0.80–1.05); pollen shapes were thus oblate-spheroidal, because the equatorial diameter (40 μm, 35–48) was generally larger than the polar length (37 μm, 33–41). Differences were observed in the polar length and equatorial diameter of the three types of pollen grains (Kruskal–Wallis test, *p* < 0.0001), with the values of both of these parameters larger in HA and M than in MA (Steel–Dwass multiple comparison, *p* < 0.01). The exine was thin, averaging 1.0 μm (0.8–1.2) thick, and its thickness did not differ among the three pollen types (Kruskal–Wallis test). The pollen sculpturing type was reticulate, and the germinal aperture was aligned with the equatorial plane. The three types of *F. platypoda* pollen grains were mainly 4-colporate, with four pores and furrows (colpi). The three pollen types only differed slightly in morphology.

#### 3.2.2. Germination Ability

In 1992, the mean germination rate of pollen grains from male trees was 40.40 ± 4.62% (*n* = 5) when tested on solid medium containing 10% sucrose and 1% agar (Table 1). In a germination test using the same tree samples and the same germination medium, the mean germination rate of pollen grains from male flowers of andromonoecious trees (15.20 ± 6.06%, *n* = 5) was higher than that from hermaphroditic flowers (5.80 ± 5.07%, *n* = 5) (χ^2^ test, *p* < 0.001). In 1995, the mean germination rate of pollen from male flowers of male trees (17.60 ± 9.66%, *n* = 5) was higher than that from male flowers of andromonoecious trees (3.00 ± 2.35%, *n* = 5) and hermaphroditic flowers of andromonoecious trees (3.00 ± 1.22%, *n* = 5) (χ^2^ test, *p* < 0.001). The germination rate of M pollen was higher than that from the other two flowers, but no difference was observed between MA and HA (Tukey’s WSD test). The pollen germination rate was higher in 1992 than in 1995. In addition, the germination rate of pollen from the male tree was higher than that of pollen from the andromonoecious tree. The germination rates of pollen from the male and hermaphroditic flowers of the andromonoecious tree were different in 1992, but the same in 1995. Thus, all pollen types were capable of germination and the germination rate varied with years, sexes, and flower types.

### 3.3. Pollination Experiments

The results of the pollination experiments indicate that *F. platypoda* may be a functional androdioecious species. In our experiments, pollen from male trees and from andromonoecious trees was viable not only ex vivo on nutrient medium, but also in vivo in recipient experimental trees (Table 2). The fruit set rates were significantly different among various treatments over the five studied years (Table 2, χ^2^ test, *p* < 0.01).

In all years, the fruit set rates from self-pollination in bagged inflorescences of andromonoecious Tree 2 and Tree X were close to zero and were the lowest among all the treatments (Tukey’s WSD test, *p* < 0.05). In the open pollination tests, Tree 2 and Tree X produced fruits in all years. The fruit set rate under open pollination fluctuated from year to year (e.g., for Tree 2: from 12.10% in 1993 to 1.85% in 2006).

Compared with the fruit set rates under open pollination, those under cross-pollination with pollen from male trees were significantly higher in 1992, 1993, and 1996 (Tukey’s WSD test, *p* < 0.05). These high rates were detected in different combinations between recipient experimental trees and pollen donor trees. The fruit set rate in Tree 2 after cross-pollination with pollen from andromonoecious trees differed between donor Tree Z and Tree 28. As indicated by the successful fruit set, Tree 2 was compatible with pollen from donor Tree 28, but incompatible with pollen from donor Tree Z.

The fruit set rate was significantly higher after cross-pollination with pollen from a male tree than after cross-pollination with pollen from an andromonoecious tree in 1996. In 1996, the mean fruit set rate with pollen from male Tree Y was 7.88% (4.44–11.23%), whereas that with pollen from andromonoecious Tree 28 was 5.16% (0–11.03%), with a large variance.

## 4. Discussion

### 4.1. Male Function of Hermaphrodites of F. platypoda

The sexual expression of the *F. platypoda* populations was clearly distinguished into two types. One type was male trees, with only male flowers with well-developed anthers and no pistils. The other type was andromonoecious trees, with hermaphroditic flowers and male flowers in the same inflorescences. Hermaphroditic flowers have a pair of anthers and a pistil [30]. These results suggest that the morphological sex expression of *F. platypoda* is not polygamous, as noted by Wallander (2013) [33], but andromonoecious. This differs from the typical androdioecious breeding system in which male trees bear only male flowers and hermaphroditic trees bear only hermaphroditic flowers. However, the sex expression of *F. platypoda* is simpler than that of *Fraxinus excelsior*, which has seven categories of sexual phenotypes [35,36]. Our findings suggest that *F. platypoda* is morphologically very close to being androdioecious, or is a kind of androdioecious tree similar to the Spanish populations of the androdioecious tree, *Phillyrea angustifolia* [16].

In the present study, male and andromonoecious trees of *F. platypoda* had identical flowering times and, other than a few size differences among pollen types, similar pollen morphologies (Figure 7). In addition, both types of flowers had dehiscent anthers. As revealed by pollen germination tests, hermaphroditic and male flowers both had viable pollen (Table 1), and both types of pollen could lead to fruit formation according to pollination experiments (Table 2). Furthermore, male trees of *F. platypoda* had unisexual flowers, and the gender of andromonoecious trees remained constant for 17 years. We thus conclude that *F. platypoda* may be a kind of functionally androdioecious tree.

According to our observations, pollen grains of *F. platypoda* are mainly 4-stephanocolporate, with four pores and furrows (colpi). Except for small differences in size, the three types of pollen in *F. platypoda* are morphologically identical.

In our study, all types of pollen were able to germinate on nutrient medium and mean pollen germination rates varied among years (Table 1). In both 1992 and 1995, pollen from male trees had a higher germination rate compared with pollen from andromonoecious trees. The germination rate of pollen from male flowers of an andromonoecious tree was higher than that of pollen from hermaphrodite flowers of andromonoecious trees in 1992, but not in 1995. In further studies, it will be necessary to extend these findings by performing a stigma test. This will clarify whether each type of pollen is compatible or incompatible [17,18,19].

Our pollination experiments resulted in the following findings. First, *F. platypoda* is self-incompatible. The mean fruit set of bagged, self-pollinated inflorescences was very low: 0% to 0.35% (Table 2), just as in *F. ornus* [27]. Second, the hermaphrodites are functionally hermaphroditic and produce viable pollen. The fruit set of hermaphrodites pollinated with other hermaphrodites was higher than that of self-pollinated plants (Table 2). Third, fruit set following natural pollination varied from year to year, with values ranging from 12.10% in 1993 to 1.85% in 2006. The high fruit set in 1993 may be attributed to the large number of male flowers in that year. In 2006, however, the fruit set was very low, even though a large number of male flowers was observed. The low fruit set in this latter case is likely due to pollination failure or abortions caused by resource limitations or very windy conditions.

In 1992, 1993, and 1996, the fruit set of flowers cross-pollinated with male pollen from male tree was higher than that following open pollination. In *F. platypoda*, this phenomenon may be indicative of pollen limitation. In natural habitats, *Fraxinus platypoda* grows in dense stands comprising a mixture of male and andromonoecious trees. Pollen limitation is not expected to occur in these forest stands because flowers of this species are wind-pollinated. The pollen limitation of *F. platypoda* inferred in this study may be due to the fact that the recipient experimental tree was far away from any male trees (Figure 1).

It has been noted that these common pollination protocols have a considerable risk of contamination [37]. The protecting bag is opened at full blooming in the forests to introduce pollen from flowers on a branch of the pollen donor tree. *F. platypoda* is anemophilous; therefore, it could be contaminated by airborne pollen during unbagged crossings. Therefore, the fruit set rates in this study are likely to be overestimated. However, the fact that the fruit set rates were higher after cross-pollination than after open pollination, even under contaminated conditions, suggests the possibility of cross-pollination effects and pollen limitation. The difference in fruit set rates between the pollen of male trees and that of andromonoecious trees under the same contaminated conditions suggests that the pollen of male trees is more effective in terms of fertilization and fruit set.

To confirm our findings and to provide more informative data, it will be necessary to control for pollen contamination with paternity analyses and use positive pollination controls of stigma receptivity [37].

### 4.2. Sexual Expression

In general, the frequency of male individuals is low in populations of androdioecious species; for example, <27% in *Datisca glomerata* [11], <30% in *Mercurialis annua* [12], and <28% in *Schizopepon bryoniaefolius* [13].

However, in both studied populations of *F. platypoda*, the frequency of males was clearly high: 50%. Such a high male frequency has also been observed in other androdioecious Oleaceae species, for example, in *Phillyrea angustifolia*, where self-incompatibility explains the high proportion of males among androdioecious plants [18]. In two groups of self-incompatible hermaphrodites, plants of each group are incompatible with each other, but are compatible with members of the other group without exception [18]. This reproductive pattern is evident in the androdioecious species *F. Ornus* [19] and the polygamous species *F. excelsior* [35]. Based on our results (notably: the pollen from andromonoecious trees is viable and leads to fruit formation) and data for other Oleaceae species, we predict that *F. platypoda* is not a cryptic-dioecious species, but a functionally androdioecious species with a DSI system.

A diallelic self-incompatibility (DSI) system could very well be the main determinant of fertilization and maintain the high frequency of males within populations of *F. platypoda,* like *F. ornus* [19]. Notably, according Wallander (2013) [33], *F. platypoda* is not very far from *F. ornus* phylogenetically. However, there are some problems in the present study: stigma tests were not performed, contamination was not checked, and comparisons were not made between different populations. These are issues that need to be addressed in the future.

Although androdioecious plants are rare [7,8,9,10,11], numerous species in Oleaceae show this sexual expression [28]. In particular, many androdioecious species are found in the genus *Fraxinus* [28]. Many of these morphologically androdioecious species are tall trees; however, their functional sexual expression is unknown. In this study, we have shown that mature *F. platypoda* trees (>30 m height) may be functionally androdioecious. The discovery of this type of sexual expression in other *Fraxinus* species is highly likely in the future. In this sense, further research on *Fraxinus* should help elucidate the evolution of sexuality in plants.

## 5. Conclusions

Finally, the DSI system could very well be the main determinant of the fertilization pattern in *F. platypoda*. This seems to be the pertinent hypothesis that needs to be verified. Are hermaphrodites distributed between two SI groups where individuals are intra-incompatible but inter-compatible? Are male individuals compatible with all hermaphrodites? The stigma test [19] should answer these two questions unambiguously.

## Figures and Tables

**Table 1:** Pollen germination rates.

Source of Pollen	1992 ***	1995 ***
Male flower of male tree (M)	40.40 ± 4.62 ^a^	17.60 ± 9.66 ^a^
Male flower of andromonoecious tree (MA)	15.20 ± 6.06 ^b^	3.00 ± 2.34 ^b^
Hermaphroditic flower of andromonoecious tree (HA)	5.80 ± 5.07 ^c^	3.00 ± 1.22 ^b^

Note: *** *p* < 0.001; ns, not significant. In the rightmost column, values followed by the same lowercase letter are not significantly different (Tukey’s WSD test, *p* < 0.05).

**Table 2:** Fruit set rates (%) in different pollination experiments in five different years (χ^2^ test; **, *p* < 0.01). Recipient experimental tree and their pollen donors (tree numbers can be found in Figure 1).

	Recipient Experimental Trees	Tree 2 1992 **	Tree 2 1993 **	Tree 2 1995 **	Tree 2 1996 **	Tree 2 2006 **	Tree X 1992U **
Pollen donor trees	Male Tree Y	12.70 ^a^	-	-	7.88 ^a^	-	15.70 ^a^
	Male Tree 1	-	14.35 ^a^	-	-	-	-
	Andromonoecious Tree Z	-	-	0.00	-	-	-
	Andromonoecious Tree 28	-	-	-	5.16 ^b^	1.06 ^a^	-
Self-pollination		0.04 ^b^	0.00 ^b^		0.30 ^c^	0.35 ^b^	0.18 ^b^
Open pollination		4.42 ^c^	12.10 ^c^	3.47	4.83 ^b^	1.85 ^c^	10.54 ^c^

Note: In each column, mean values followed by different lowercase letters are significantly different (Tukey’s WSD test, *p* <0.05). Experiments were conducted on the same single individual (Tree 2), except for 1992U.
